# The Impact of Brain Breaks Classroom-Based Physical Activities on Attitudes toward Physical Activity in Polish School Children in Third to Fifth Grade

**DOI:** 10.3390/ijerph15020368

**Published:** 2018-02-21

**Authors:** Agata Glapa, Joanna Grzesiak, Ida Laudanska-Krzeminska, Ming-Kai Chin, Christopher R. Edginton, Magdalena Mo Ching Mok, Michal Bronikowski

**Affiliations:** 1 University School of Physical Education in Poznań, Królowej Jadwigi 27/39, 61-871 Poznań, Poland; joanna.grzesiak2904@gmail.com (J.G.); idalk@poczta.fm (I.L.-K.); bronikowski.michal@wp.pl (M.B.); 2 HOPSports, Inc., 4262 Blue Diamond Road #102-359, Las Vegas, NV 89139, USA; mingkai@hopsports.com; 3 University of Northern Iowa, 105 Human Performance Center, Cedar Falls, IA 50614, USA; christopher.edginton@uni.edu; 4 Assessment Research Centre, and Department of Psychology, The Education University of Hong Kong, 10 Lo Ping Rd, Taipo, Hong Kong; mmcmok@eduhk.hk

**Keywords:** primary schools, children, physical activity, video games, exercise, Brain Breaks^®^

## Abstract

The purpose of this study was to examine the effectiveness of the Brain Breaks^®^ Physical Activity Solutions in changing attitudes toward physical activity of school children in a community in Poland. In 2015, a sample of 326 pupils aged 9–11 years old from 19 classes at three selected primary schools were randomly assigned to control and experimental groups within the study. During the classes, children in the experimental group performed physical activities two times per day in three to five minutes using Brain Breaks^®^ videos for four months, while the control group did not use the videos during the test period. Students’ attitudes toward physical activities were assessed before and after the intervention using the “Attitudes toward Physical Activity Scale”. Repeated measures of ANOVA were used to examine the change from pre- to post-intervention. Overall, a repeated measures ANOVA indicated time-by-group interaction effects in ‘Self-efficacy on learning with video exercises’, *F*(1.32) = 75.28, *p* = 0.00, η^2^ = 0.19. Although the changes are minor, there were benefits of the intervention. It may be concluded that HOPSports Brain Breaks^®^ Physical Activity Program contributes to better self-efficacy on learning while using video exercise of primary school children.

## 1. Introduction

Physical activity (PA) plays an important role in neuromuscular development, energy balance, and obesity prevention among children during their early years. Recent evidence [1,2,3] suggests that an optimal level of PA can positively influence cortical and sub-cortical brain neurogenesis, neuromuscular system maturation, primitive reflex and stereotypical postures reduction, improvement of concentration, planning, coordination processes and increase of executive control, on-task behavior and academic performance. Reduction of PA can lead to increase of chronic health diseases also leading to negative physiological, cognitive and behavioral outcomes. Increasing sedentary behavior patterns among children can be a crucial factor causing reduced activity time leading to prevalence of overweight and obesity [4]. Reasons for the decrease in PA of children are ascribed to the effects of environmental factors, i.e., development of technologies coupled with excessive use of social networks and communication programs on the Internet (amount of time children spend watching television and sitting in front of the computer), extra-curricular activities at the desk, lessons or homework and a decrease of community contacts [5]. An effort to halt or reverse these trends through promotion of PA among children and adolescents has been identified as a key focus of efforts to promote health and encourage habits that reinforce participating in PA [1].

There is a need to initiate PA programs which promote curiosity and fun during this time of intensified development (6–12 years old). Research suggests that participation in PA can offer individuals an opportunity to develop a “cheerful and joyous spirit” and provide for a source of enjoyment [6]. Development between 6 and 12 years is recognised as a “golden period of motor development” when children acquire most of habits which are used in adulthood [7]. During this period, physical characteristics of physical growth, cognitive and motor development are developing.

This process is connecting with complex motor skills acquisition, locomotor coordination development and the organisation in cognitive and in the executive cerebral cortex area of the brain [8,9,10]. It is during this time that children start their school education and the process of physical literacy slows down due to new educational responsibilities. Therefore, the importance of connecting cognitive development in first years at school with adequate level of PA necessary to maintain the appropriate development, tempo and direction is critical [11].

Additionally, recommended levels of PA for children aged 6–18 years [12] call for accumulation of at least 60 minutes a day of moderate to vigorous intensity. Only some of it may be covered through school physical education (PE) lessons [13]. Therefore, the important need is to find new ways to promote PA and encourage behaviour change to increase participation in PA among children by making it interactive, fun and engaging in the school environment. Recent investigations showed that although playing computer games causes a sedentary life, technology can be also used effectively to promote active lifestyles [14,15]. In this age of technology, our children are thinking and functioning differently to any previous generations. Interactive video games and internet-based PA interventions became more attractive and effective for children with the improvement of PA in school setting or in extra-curricular environments [16,17,18]. A comprehensive review of research on implementation of school-based PA programs report either positive or non-significant enhancement of cognitive skills and attitudes and academic performance with only few investigations demonstrating negative relationship [19].

Therefore, the development and evaluation of interventions to promote PA in children ought to be a priority. A promising venue for the improvement in daily PA of school pupils comes through school recess or other extra-curricular activities [20]. Since 1980, schools have been identified as ideal settings for the promotion of health and PA behaviors as they offer great potential to intervene because of the considerable portion of walking time for the child. School also provides an environment where healthy habits are formed [21]. Studies suggest [22,23] that high-quality PE, with appropriately high-intensity profiles is linked to an increase in after-school leisure time PA. School-based interventions aiming at increasing PA bring promising results [24,25] when accompanied with active parental involvement [26,27,28].

PA with community involvement and educational interventions when paired with school policy and environmental changes is likely to be effective among children and adolescents and should be promoted. Therefore, a multilevel approach to promoting PA, combining school-based interventions; use of modern technology to encourage an active lifestyle seems to be a good idea to overcome time constraints and other environmental challenges of educational system [15,29].

An intervention to meet the above-mentioned goals is school-based—a video-exercise intervention called Brain Breaks^®^ Physical Activity Solutions by HOPSports^®^ [30]. The Brain Breaks^®^ program could become one of the effective strategies to increase children’s levels of PA in school environments, reaching a wide number of children who are spending most of their time in schools [23]. Brain Breaks^®^ Physical Activity Solutions are web-based structured PA breaks that stimulate students’ interest in learning and in the promotion of health and wellness. The short videos (three to five minutes during the teacher-ordered break between lessons) are specifically designed for the classroom settings to motivate students to enhance their PA at theoretical lessons and provide a platform not only to be physically active during breaks, but also learn new motor skills, coordination, dance, movements of highly integrated functional muscles groups in movement activity, language, art, music, and different cultures [26].

As such, the aim of the study was to investigate the effects of providing the Brain Breaks^®^ Physical Activity Solutions during the school lessons on children’s changes in attitudes toward PA over a four-month intervention program. In the present study, it was hypothesized that the Brain Breaks^®^ Physical Activity Solutions may change the attitude toward beliefs, self-efficacy, self-confidence and motivation on the PA of school children.

## 2. Methods

### 2.1. Study Design and Participants

Participants included 326 primary school children (170 boys and 156 girls) aged 9–11 years (9.74 ± 1.06 years) from 3rd to 5th grade (Table 1). In total 19 classes from 3 randomly selected public schools in a community in Poland were recruited. All students in the recruited classes were invited to participate. Four hundred students agreed to participate in the survey and complete questionnaires (response rate was 81.5%). Seventy-four students were excluded from the analysis due to an incompletely filled out questionnaire. Therefore, the final sample consisted of 326 students. A two-group pretest/posttest study was conducted. An intervention was designed with experimental (*n* = 264) and control group (*n* = 62) from each school. The classes were randomly assigned to these groups (with a similar number of pupils from each grade).

Prior to intervention implementation (September–November 2014), a trained research assistant visited each school and introduced the study procedure. In general, the intervention was run for 4 consecutive months, from January to April 2015 (including an Easter holiday and winter break). During implementation, teachers involved in the program used the digital platform provided by HOPSports. The HOPSports is a technology system that is used in a class or group environment that allows participants to follow an on-screen instructor leading an activity. The Brain Breaks^®^ Physical Activity Solutions is a set of 3–5 minutes videos, based on PA during breaks during the school day. All activities are designed as part of a standard-based lesson plan in order to develop a particular skill of fitness component. Online access to the official project website is found at: http://hopsports.com/what-is-brain-breaks was available at all times.

The teachers were instructed by the research assistant on how to implement the intervention and received an individual access to the platform. Teachers had to choose a 3–5 minutes video to be performed by children 2 times per day. Furthermore, those teachers were required to complete daily and monthly reports showing the number of videos taken per month in each of the selected classes. Before and after implementing the program, data on children’s PA behaviours and self-report of participation in the program were collected using an anonymous questionnaire survey. The questionnaires were numbered using a code for matching the pretest and posttest data. The control group did not have breaks with Brain Brakes videos even though students in the control group also completed the questionnaire survey at the same time as that of the experimental group. The survey questionnaire took about 30 minutes to complete in quiet classroom conditions.

### 2.2. Self-Report Physical Activity Behavioural Measures

The Attitude toward Physical Activity Scale (APAS) was used to measure children’s attitudes and perceptions regarding various aspects of engagement in PA, with particular emphasis on exercising using video games. The APAS questionnaire consists of a demographic section (gender, age, grade, body height and weight) and six other sections. The sections corresponded to six scales, namely: (1) ‘Promoting holistic health’: a 10-item scale constructed to measure students’ attitudes toward the effectiveness of physical activities in promoting holistic health; (2) ‘Importance of exercise habit’: a 5-item scale designed to measure students’ attitude toward the importance of doing exercise as a lifestyle; (3) ‘Self-efficacy in learning with video exercises’: a 15-item scale constructed to measure students’ self-efficacy in learning curriculum subjects by using video exercises; (4) ‘Exercise motivation and enjoyment’: a 14-item scale designed to measure students’ motivation for and enjoyment in doing physical exercise; (5) ‘Self-confidence on physical fitness’: an 8-item scale constructed to measure students’ self-perception of physical fitness; (6) ‘Trying to do personal best’: a 5-item scale constructed to measure students’ personal best goal orientation to engage in physical activity.

Responses to the items were on a 4-point Likert scale ranging from 1 (“Strongly disagree”), 2 (“Disagree”), 3 (“Agree”) to 4 (“Strongly agree”). The original English version of the questionnaire had been validated in a previous study [31] using Rasch analysis which provided empirical support to the reliability, unidimensionality, effectiveness of response categories, and absence of gender differential item functioning (DIF) of the scales. In this study, the questionnaire items were firstly inspected for cultural appropriateness. They were then translated from English to Polish with back translation, and the translated version was adjusted by the researchers before the questionnaire was administered. In our study, the scale’s internal consistency established within Cronbach’s alpha test was 0.92 (with 0.69 for ‘Promoting the holistic health scale’, 0.53 for ‘Importance of exercise habit scale’, 0.95 for ‘Self-efficacy in learning with video exercises scale’, 0.86 for ‘Exercise motivation and enjoyment scale’, 0.88 for ‘Self-confidence on physical fitness scale’ and 0.90 for ‘Trying to do personal best scale’).

### 2.3. Ethics

All study procedures were reviewed and approved by institutional review board. All participants took part in the study voluntarily and could discontinue their participation at any time. Prior to study enrollment, all study participants provided written informed assent in conjunction with parental written informed consent. The research protocol was approved by the Ethical Committee of the Local Bioethics Committee of the Karol Marcinkowski University of Medical Sciences in Poznan (decision no.1080/12). All personal data were anonymised.

### 2.4. Statistical Analysis

The Statistica software program (version 12.0, StatSoft, Krakow, Poland) was used to process the data. Descriptive statistics (means, standard deviations and frequencies) were used to describe the sample. An independent samples *t*-test was conducted to compare the groups (experimental vs. control) at the baseline of the intervention for all variables. Changes in analysed variables from pretest to posttest were assessed using repeated measures analysis of variance (ANOVA) to determine TIME and TIME-by-GROUP differences. To conduct multiple comparisons, Tukey’s posthoc test for unequal sample size was used. To study the development (or change) over time of all variables, Pearson’s correlation coefficients were calculated for pretest and posttest. The correlation effect size (*r*) was calculated. Guideline values of correlation effect size *p* = 0.1, 0.3, and 0.5 were regarded as small, medium and large effects respectively [32]. Results were considered significant at *p* < 0.05.

## 3. Results

There were no significant differences in investigated variables between groups at baseline of the intervention. When analysing the pretest mean scores in each of the scales, the highest ones were: ‘Importance of the exercise habit’, ‘Trying to do personal best’, ‘Self-confidence on physical fitness’, ‘Exercise motivation and enjoyment’, ‘Promoting the holistic health’, and ‘Self-efficacy in learning with video exercises’, for both groups.

For the control group, all scales with the exception of one decreased in their mean values from pretest to posttest. The exception was the ‘Self-confidence on physical fitness’ scale, which did not change its mean value from pretest to posttest. For the experimental group, three scales, namely, the ‘Promoting the holistic health’ scale, the ‘Self-efficacy on learning with video exercises’ scale, and the ‘Exercise motivation and enjoyment scale’, increased their means from pretest to posttest while the remaining three scales did not change their values from pretest to posttest. Nevertheless, not all the increases reached statistical significance. Repeated measures ANOVA indicated a significant TIME interaction effect for the ‘Trying to do personal best’ scale (*F*(1,32) = 4.92, *p* = 0.03, *ƞ*^2^ = 0.01) (Table 2). The results also indicated a significant TIME x GROUP interaction effect in Self-efficacy on learning with video exercises’ (*F*(1,32) = 75.28, *p* = 0.00, *ƞ*^2^ = 0.19). Tukey’s post hoc test indicated the difference between means of ‘Self-efficacy on learning with video exercises’ after the intervention among two groups (*p* < 0.01). None of the other scales showed any TIME or TIME × GROUP interaction effect.

**Figure 1:**
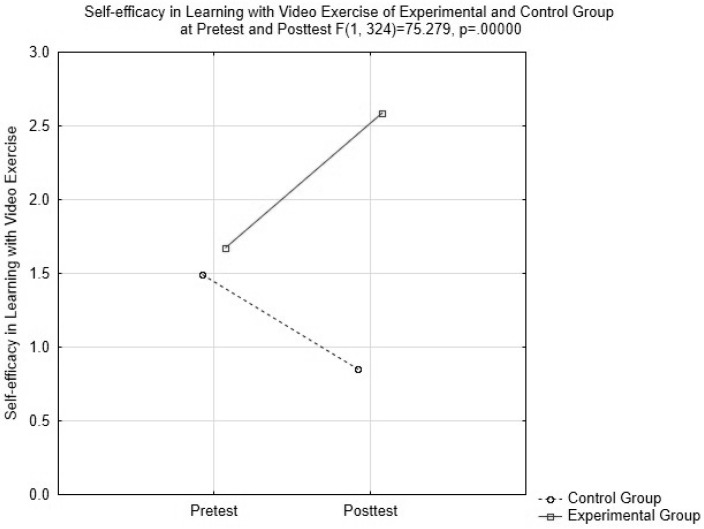
Distribution of the ‘Self-efficacy on learning with video exercises’ scale scores for the Experimental and Control groups at pretest and posttest.

As illustrated in Figure 1, whilst the experimental and control group were similar in scores in ‘Self-efficacy on learning with video exercises’ at pretest, the experimental group gained significantly more than the control group from pretest to posttest, resulting in the experimental group having substantially higher scores than the control group in posttest.

## 4. Discussion

The primary objective of this study was to investigate the effects of providing the Brain Breaks^®^ Physical Activity Solutions during the school breaks between lessons on children’s changes in attitude toward PA over a four-month intervention program. The effectiveness of an intervention is usually evaluated through the changes in the mean values of the intervention group representing progress or regress of particular variables.

In the present study, the hypothesis was confirmed only in one variable. Classroom exercise breaks improved students’ ‘Self-efficacy in learning with video exercises’ in experimental group. The Brain Breaks activities intervention contributed to the children’s development of health-related areas of knowledge including health, healthy lifestyle, and healthy diet, and also some specific academic knowledge in the areas of language, mathematics, art, writing, music, culture, composition, and environmental protection. Similar to the current study, a previous study where the same scale was used also found positive effects in ‘Self-efficacy in learning with video exercises’ after the four-month intervention program [33]. While the control group (the group without receiving the Brain Breaks activities) had decreased ‘Self-efficacy in learning with video exercises’ and ‘Trying to do personal best’, the decreases were statistically significant. In a Turkish study [33], only small point increments were reported in each scale, which was not significant for the control group.

In the study of Reilly, Buskist and Gross [34] it was found that movement in the classroom boosts brain power and that regular PA breaks given during the school day had a positive impact on primary school students academic performance and behavior in the classroom. This finding is supported by studies showing the positive associations between PA and academic performance [35]. The literature is supportive of the concept of using activity programs including significant amounts of PA in the classroom such as brain breaks to promoting increased levels of PA of children [36,37], for the improvement of cognition, attention, motivation, on-task behavior and academic outcomes [1,3,38,39]. PA has been demonstrated in studies to increase blood flow to the brain with an increase in oxygen level which has the potential to have an impact on brain function [40,41].

Breaks during a lesson have also had a positive impact on learners’ motivation and achievement and on student’s enjoyment during learning, their motivation for learning and their focus [42]. There is even some evidence that PA breaks that include an academic component can improve time on-task in primary school pupils [43].

An active video game may provide a more popular option for lifetime PA than traditional exercising and sports [44,45]. According to Warburton et al. [46] the mass appeals of video games among children, youth and young adults may allow this form of intervention to become a useful tool to promote PA. It is also worth noting that the interactive system does not produce the level of competition and is catered to everyone, including the obese, overweight and inactive children with equal opportunity. There is no competition involved in providing all students extra PA. All students are encouraged to focus their energy on self-improvement. The benefit of non-competitive and individually-based PA was noticed also in the results of our study, where children from the control group indicated lower results in ‘Trying to do personal best’, which might be linked to the classes of PE, where children have more opportunities for comparing with other peers. In turn, during Brain Breaks activities they are focused entirely on themselves and on a task. Positive PA experiences have been shown positively related with the associated physical self-concept [6].

As for the lack of improvement in other areas of attitudes toward PA (for example, ‘Exercise motivation and enjoyment’, ‘Self-confidence on physical fitness’, ‘Promoting the holistic health’ or ‘Importance of exercise habit’), it might be attributed to the Polish education system. Polish schools have mostly the primary priority to improve cognition as they are oriented on academic achievements (lots of standardized achievement tests) and are also under pressure to improve academic scores, therefore it might be that teachers spend additional time for cognitive learning and less time for physically active classes. Adding PA to the school day can be difficult due to the competing priorities and lack of time reported by teachers or school administrations [47]. This problem is not limited to Poland; the recent cutting of school PE time in the State of Illinois (USA) for reading and math instruction has been reported [48]. However, previous studies have demonstrated that increases in time spent on PA are not likely to detract from students’ academic effort [1,19]. Therefore, classroom teachers should be encouraged to devote time during academic learning to incorporate PA.

The intervention was implemented by different teachers, which might also have affected the results of our study. According to Lagarde et al. [49], regardless of which method used to increase PA in schools, classroom teachers are critical stakeholders for the success of the program and bringing them on board is not an easy task. The findings regarding the impact of classroom activity breaks also suggested that the classroom teachers would play an important role of influence in the daily activity patterns of children [33]. Teachers need to be trained to implement these practices. Future trainings preparing teachers to implement the intervention should help them to perform the same set of the classroom exercise breaks without help of the research staff. Efficient and effective PA professional development for teachers is needed. Furthermore, teachers’ perception of classroom PA breaks is important. They can identify barriers in implementation of this kind of intervention. Caldwell and Ratliffe [50] also suggested brief messages on the benefits of regular exercise explained by the teachers on the reasons of participating in the classroom PA would be beneficial to the children in addition to performing the exercises.

There is a need to highlight that the background of the Brain Breaks^®^ intervention is a focus on fun and enjoyment. Studies show that the level of PA is proportionally higher when children are given the opportunity to play active games and experience fun [51]. Therefore, although the changes after the experimental program are mild, there are some additional benefits of the intervention. The HOPSports Brain Breaks^®^ Physical Activity Program contributes to better self-efficacy on learning while using video exercise with primary school children.

Some strengths and limitations of this study are worth noting. The most important strength is the experimental nature/design of the study. Another strength of the present study was the ability to study effectiveness of real-world intervention on pupils attitude toward PA. A strength is also the number of the sample size. Furthermore, according to our knowledge, this is the first time this type of research on Brain Breaks^®^ Physical Activity Solutions was conducted among children in Poland. These results should therefore provide a useful basis for future research. A limitation is the self-reporting nature of the surveys and the strict curricula and limited time in primary schools. Administrators and teachers must both be flexible to integrate PA opportunities throughout the school day. Finally, the intervention study as a whole would have been stronger if we had been able to include an objective measure of students PA level (for example with pedometers or accelerometers).

The researchers of this study believe that the research provides some initial evidence of the potential gains of using Brain Breaks^®^ activities. These are preliminary analyses and more research is needed to understand how regular classroom exercises—so-called ‘brain breaks’—will affect students’ attitudes toward beliefs, self-efficacy, self-confidence and motivation on the PA of school children. Future studies are needed with a larger and more diverse population with comparison to support these findings.

## 5. Conclusions

The results of this study showed that a school-based Brain Breaks^®^ activities intervention program designed to make children more physically active during the school day significantly improved mainly the children’s ‘Self-efficacy on learning while using video exercise’. The most significant value of HOPSports Brain Breaks^®^ Physical Activity Solutions may be its provision of physically active time in the lessons that are relatively easy to facilitate. Therefore, the value of Brain Breaks^®^ activities might more depend on the teachers’ experience, as well as teacher ability to plan engaging, physically active lessons, than on the technology itself.

## Figures and Tables

**Table 1:** General characteristics (mean, standard deviation and frequency) of the participants.

Variables	Total *n* = 326	CG *n* = 62 (19.0%)	EG *n* = 264 (81.0%)
Age (years)	9.7 ± 1.06	10.1 ± 0.92	9.6 ± 1.08
Body height (cm)	144.0 ± 9.17	146.0 ± 9.39	143.5 ± 9.07
Body weight (kg)	35.5 ± 6.92	37.8 ± 8.62	34.9 ± 6.35
Gender			
Male	170 (52.1%)	38 (61.3%)	132 (50.0%)
Female	156 (47.9%)	24 (38.7%)	132 (50.0%)
Grade level			
Grade 3	107 (32.8%)	20 (32.2%)	87 (33.0%)
Grade 4	109 (33.4%)	20 (32.2%)	89 (33.7%)
Grade 5	110 (33.8%)	22 (35.6%)	88 (33.3%)

Notes: CG = Control Group; EG = Experimental Group.

**Table 2:** Descriptive statistics and results of ANOVA variables before and after the intervention programme (pretest vs. posttest) in Experimental Group (*n* = 264) and Control Group (*n* = 62).

Variables (pts)	Group	Pretest M ± SD	Posttest M ± SD	Time		Time x Group	
				*F* (*p*)	*ƞ*^2^	*F* (*p*)	*ƞ*^2^
Promoting the holistic health	EG	3.0 ± 0.56	3.1 ± 0.60	0.79 (0.37)		2.89 (0.09)	
	CG	2.9 ± 0.47	2.8 ± 0.63				
Importance of exercise habit	EG	3.3 ± 0.81	3.3 ± 0.67	0.94 (0.33)		0.48 (0.49)	
	CG	3.3 ± 0.56	3.2 ± 0.68				
Self-efficacy on learning with video exercises	EG	1.7 ± 1.03	2.6 ± 0.71	2.22 (0.14)		75.28 (0.00)	0.19 **
	CG	1.5 ± 0.86	0.8 ± 0.75				
Exercise motivation and enjoyment	EG	3.0 ± 0.58	3.1 ± 0.65	0.58 (0.45)		1.52 (0.22)	
	CG	2.9 ± 0.47	2.8 ± 0.75				
Self confidence on physical fitness	EG	3.1 ± 0.69	3.1 ± 0.77	0.00 (0.98)		0.18 (0.67)	
	CG	2.9 ± 0.73	2.9 ± 0.85				
Trying to do personal best	EG	3.2 ± 0.77	3.2 ± 0.87	4.92 (0.03)	0.01*	2.26 (0.13)	
	CG	3.2 ± 0.58	2.9 ± 0.99				

Notes: EG = experimental group, CG = control group; * *p* < 0.05, ** *p* < 0.01.
